# Mobilization of the high-riding vertebral artery for C2 screw insertion

**DOI:** 10.3171/2020.4.FocusVid.20172

**Published:** 2020-07-01

**Authors:** Atul Goel

**Affiliations:** Department of Neurosurgery, K.E.M. Hospital and Seth G.S. Medical College, Parel, Mumbai, India

**Keywords:** atlantoaxial fixation, atlas vertebra, axis vertebra, vertebral artery

## Abstract

The basic surgical steps in the Goel technique of atlantoaxial fixation involve exposure of the atlantoaxial articulation, denuding of the articular cartilage, stuffing of bone graft pieces within the articular cavity, and subsequent instrumentation.

“High-riding” vertebral artery in relationship to the pedicle-facet of C2 has been widely recognized to be a factor that makes insertion of the C2 pedicle-facet screw difficult or impossible. In this video, a technique of exposure and mobilization of the high-riding vertebral artery to permit safe C2 screw insertion is shown. An alternative option in the presence of such a high-riding vertebral artery is to use either C2 laminar or inferior facetal screw insertion.

The video can be found here: https://youtu.be/LjxxINmzph0

## Transcript

I wish to demonstrate to you the technique of mobilization of high-riding vertebral artery into the C2 pedicle and facet to facilitate C2 screw insertion in the Goel’s technique of atlantoaxial lateral mass plate and screw stabilization.

**0:40** Clinical summary. This is a case of a 10-year-old girl who sustained blunt injury on the head 6 weeks prior to admission.

**0:48** Preoperative investigations. Investigations showed os odontoideum and mobile atlantoaxial instability. CT scan showed high-riding vertebral artery that deeply indented into the pedicle facet of C2 vertebra, more on the right side (Cacciola et al., 2004; Goel et al., 2020a).

**1:08** Position. The patient is placed in head high position under cervical traction as shown in the figure.

**1:15** Surgical technique. Midline posterior incision is taken that is centered on the C2 spinous process. Dissection is done to expose subperiosteally the lamina and pedicle of C2. The technique of atlantoaxial fixation is shown on the left side where the vertebral artery is not so high. Atlantoaxial facetal articulation is exposed by subperiosteal dissection. The facet of C1 is widely exposed. C2 ganglion is displaced rostrally. Part of the pars and pedicle of C2 vertebra are drilled to facilitate positioning of the plate for plate and screw fixation technique. The joint cavity is exposed widely as seen here and power-driven drill is used to make a hole in the facet of atlas. Bone graft is packed into the atlantoaxial facetal articulation. Screw is first placed over the plate into the C1 facet. The vertebral artery in the region of the pedicle is identified, its course is delineated, and a screw insertion point on the pedicle is identified that is away from the loop of the vertebral artery. Navigation can be used in this instance if there is significant doubt of the course of the vertebral artery. After making a guide hole, the screws are inserted and later tightened (Goel et al., 2002; Goel and Laheri, 1994).

The dissection is now done on the right side where the vertebral artery is significantly high riding. The lamina is first exposed subperiosteally. The pedicles are identified. The course of the vertebral artery is identified and exposed. The vertebral artery is seen under the suction just below the atlantoaxial facetal articulation. A hole is made into the C1 facet. Bone graft is packed into the articulation as on the left side. Bone graft is packed as much as it is possible. This packing of bone graft is absolutely crucial for bone healing and also distraction and reduction of the atlantoaxial instability. Bone is removed on the posterior surface of the vertebral artery as much as its medial extension to facilitate its mobilization. As you can see, the vertebral artery is pulsating under the hole of the C2 portion of the plate. After protecting the vertebral artery, a hole is made into the C2 facet. C2 screw is then inserted after protecting the vertebral artery away from the site of screw insertion (a small spatula or retractor can be used to protect the vertebral artery during screw insertion). The screws are then tightened (Goel et al., 2020b).

The arch of atlas and the C2 spinous process are denuded off all their muscle attachment and are drilled to make them suitable as a host bone for bone graft. Bone graft is harvested from the iliac crest.

**10:13** Postoperative imaging. Postoperative imaging shows reduction of the atlantoaxial instability and lateral mass plate and screw fixation. The C2 screw avoids the vertebral artery.

## References

[1] Cacciola F Phalke U Goel A Vertebral artery in relationship to C1-C2 vertebrae: an anatomical study Neurol India 2004 52 178 184 15269464

[2] Goel A Desai K Muzumdar D Atlantoaxial fixation using plate and screw method: a report of 160 treated patients Neurosurgery 2002 51 1351 1357 12445339

[3] Goel A Laheri VK Plate and screw fixation for atlanto-axial dislocation Acta Neurochir (Wien) 1994 129 1–2 47 53 7998495 10.1007/BF01400872

[4] Goel A Patil A Shah A Os odontoideum: analsis of 190 surgically treated cases World Neurosurg 2020a 134 e512 e523 31669688 10.1016/j.wneu.2019.10.107

[5] Goel A Rangnekar R Shah A Mobilization of the vertebral artery—surgical option for C2 screw fixation in cases with “high riding” vertebral artery Oper Neurosurg (Hagerstown) 2020b 18 6 648 651 31555817 10.1093/ons/opz289

